# Herbal medicine use behaviour in Australian adults who experience anxiety: a descriptive study

**DOI:** 10.1186/s12906-016-1022-3

**Published:** 2016-02-11

**Authors:** Erica McIntyre, Anthony J. Saliba, Karl K. Wiener, Jerome Sarris

**Affiliations:** Charles Sturt University, Wagga Wagga, NSW Australia

**Keywords:** Herbal medicine, Anxiety, Complementary medicine usage, Self-care, Disclosure

## Abstract

**Background:**

Anxiety disorders are the most prevalent mental health condition in Australia. In addition, there are many people who experience problematic anxiety symptoms who do not receive an anxiety disorder diagnosis but require treatment. As herbal medicine use is popular in Australia, and little is known about how adults experiencing anxiety are using these medicines, this study aimed to identify how Australian adults who experience anxiety are using herbal medicines.

**Methods:**

An online cross-sectional descriptive study was conducted using purposive convenience sampling to recruit Australian adults who have experienced anxiety symptoms and have used herbal medicines (*N* = 400). Descriptive statistics, chi-square test of contingency, analysis of variance, and simple logistic regression was used to analyse the data.

**Results:**

Eighty two percent of participants experienced anxiety symptoms in the previous 12 months, with 47 % reporting having previously been diagnosed with an anxiety disorder. In addition, 72.8 % had used herbal medicines specifically for anxiety symptoms in their lifetime, while 55.3 % had used prescribed pharmaceuticals, with 27.5 % having used herbal medicines concurrently with prescribed pharmaceuticals. The Internet and family and friends were the most frequently used sources of information about herbal medicines. Forty eight percent of participants did not disclose their herbal medicine use to their doctor.

**Conclusions:**

Herbal medicines are being used by adults with anxiety and are commonly self-prescribed for anxiety symptoms. Health practitioners who are experts in herbal medicine prescribing are consulted infrequently. In addition, herbal medicine use is often not disclosed to health practitioners. These behaviours are concerning as people may not be receiving the most suitable treatments, and their use of herbal medicines may even be dangerous. It is critical we develop a better understanding of why people are using these medicines, and how we can develop improved health literacy to help with treatment decision making to ensure people receive optimal care.

## Background

The experience of anxiety is complex, and anxiety disorders are the most prevalent mental health condition in Australia. Over a quarter of Australian adults reported having an anxiety disorder diagnosis in their lifetime, and 14.4 % had a diagnosis in the previous 12 months [1]. These figures exclude many people who experience problematic anxiety symptoms but do not meet the diagnostic criteria of an anxiety disorder [2, 3], thus is likely to underestimate prevalence. Both psychological and pharmaceutical evidence-based treatments are used to treat anxiety symptoms with varying levels of success. People can have poor treatment compliance, find treatments ineffective, or be concerned about the unwanted side-effects of pharmacotherapies [4]. In addition, people may not seek treatment through health professionals due to fear of stigma or lack of accessibility [5]. This may lead people to seek out alternative or complementary treatments such as herbal medicines, which are widely available to the public and allow for self-prescription.

Herbal medicines are a popular complementary and alternative medicine (CAM) in Australia, with recent prevalence in the general population estimated at 37 % [6]. However, little is known about herbal medicine use in adults who experience anxiety. A recent review of the literature found that herbal medicine use for anxiety symptoms ranged from 2 % (general population sample) to 22 % (of those with an anxiety disorder diagnosis) across four countries [7]. This review also found that people with more severe anxiety (i.e. an anxiety disorder diagnosis) were significantly more likely to use herbal medicines compared to those with less severe anxiety (i.e. no disorder diagnosis) [8, 9]. In addition, people with a generalised anxiety disorder (GAD) diagnosis (*Diagnostic and Statistical Manual of Mental Disorders-IV*) used more herbal medicines compared to those with any other anxiety disorder diagnosis [10, 11].

While herbal medicines may complement conventional treatments, or provide an effective alternative for some people, there are potential problems with the use of herbal medicines. For example, there is a risk of herb-drug interactions if taking pharmaceutical medicines concurrently with herbal medicines [12]. There is also a risk that those who are self-prescribing may be using ineffective treatments, or may not be receiving the most suitable treatments [13]. This is supported by research revealing that people using herbal medicines do not necessarily take them consistent with their evidence-based indications. Bardia and colleagues [14] found that only 54.9 % of herbal medicine users in the US used herbal medicines in line with their evidence-based indications. The authors suggest that the correct information on the indications for herbal medicines may not be reaching consumers. One explanation for this may be that people are getting their herbal medicine information from non-professional sources such as friends and family, the Internet, or popular media. High use of non-professional information sources for herbal medicines has been reported in an Australian study of pregnant woman, with 32 % of herbal medicine users (*n* = 458) self-prescribing them for anxiety symptoms [15], but it is unclear how this relates to information sources in the general population.

The potential risks of herbal medicine use are increased if people do not disclose their herbal medicine use to health practitioners. Non-disclosure of CAM use was found to be as high as 37 % in Australian psychiatric patients (*N* = 52) [16]. Research on a range of cohorts has found high non-disclosure rates of CAM use to health practitioners for fear of discrimination, or beliefs that their CAM use is not relevant to the practitioner, or that the practitioner did not ask about their CAM use [17]. The non-disclosure rates of herbal medicine use in adults experiencing anxiety is yet to be reported.

As there is no research to date that has reported the herbal medicine use behaviour of adults experiencing anxiety, the overall aim of this study was to identify how Australian adults who experience anxiety are using herbal medicines. As people with an anxiety disorder diagnosis have been found to have greater use of herbal medicines than those without a diagnosis [8], we hypothesised:

1. Having an anxiety disorder diagnosis would predict a greater probability of herbal medicine use for anxiety symptoms in the previous 12 months compared to those without a disorder diagnosis
2. Having more severe anxiety symptoms in the previous week would predict greater current herbal medicine use for anxiety symptoms than people with less severe symptoms

## Methods

### Recruitment and participants

Purposive criterion sampling was used to recruit Australian adults (18 years and over) who have used herbal medicines, and have experienced anxiety symptoms in their lifetime. Anxiety symptoms were described to participants as sweating, palpitations, nervousness, trembling, muscular tension, restlessness, feeling easily fatigued, irritability, over reaction to surprises, difficulty concentrating, irrational fears, worry, and sleep disturbances. Herbal medicines were defined as medicines made from whole plant parts in the form of tablets, capsules, liquid extracts, teas, decoctions, creams and ointments.

Survey invitations were emailed to 10,575 members of an existing database representative of the general Australian population. The database consisted of people who had registered their interest in participating in research. Eight hundred and ninety six people responded to the invitation—a response rate of 8.47 %. Of the 896 respondents 62.2 % (*n* = 544) had used herbal medicines, and 79.6 % (*n* = 710) had experienced anxiety symptoms. Four hundred people met the criteria for the study (i.e. used herbal medicines and had experienced anxiety).

### Procedure

Ethics approval was provided by Charles Sturt University (2014/033) in accordance with the declaration of Helsinki.

Once participants responded to the emailed invitation they were directed to a GroupQuality® online survey tool where participants could complete the survey. An information page was presented to participants that asked for informed consent. Continuing to complete the survey was considered consent. They were then required to answer *yes* or *no* to three screening questions: 1) Are you 18 years of age or older?; 2) To the best of your knowledge, have you ever experienced anxiety?; and 3) Have you ever used herbal medicines? If answering *no* to any of these questions they did not meet the criteria, were thanked for their time, and could not continue with the survey. The survey took approximately 30 min to complete. Participants were paid a nominal amount for their participation based on time taken to complete ($10 for 30 min).

### Measures

#### Demographic and medicine use questionnaire

A questionnaire was used that included the demographic items: age, gender, postcode, and education level; and items measuring herbal and pharmaceutical medicine use, communication with health care providers, information sources use and disclosure of herbal medicine use.

#### Health practitioner and herbal medicine use questions

Participants were asked which health providers they had seen in the previous 12 months from a list of practitioners who were considered most likely to prescribe herbal medicines; they also had the opportunity to indicate “other” if there were practitioners they had seen that were not on the list. Both the practitioner use questions and the herbal medicine use questions were adapted from the International Complementary and Alternative Medicine Questionnaire [18], which measures CAM use. The changes made to the questionnaire were related to formatting to enable improved online delivery, and to focus specifically on herbal medicines rather than CAM in general. The question *Have you used any of these herbs in the last 12 months?* was presented to participants with the option of choosing from a list of 17 herbs commonly used to treat anxiety, plus the option of choosing “herbal formula” or “other herbs used”. For each herb selected a series of questions were asked relating to how the medicines were used, which included current use of each herb selected. A variable for current herbal medicine use was created, with 2 = *Yes* if participants indicated they currently used at least one herbal medicine for anxiety, and 1 = *No* if they did not currently use a herbal medicine for anxiety. The item *During the last 12 months I have used herbal medicines to treat anxiety symptoms* was measured using a Likert type scale ranging from 1 = False and 7 = True.

#### Anxiety disorder diagnosis

A dichotomous (*Yes, No*) item was used to measure self-reported anxiety disorder diagnosis: *Have you ever been diagnosed with an anxiety disorder?*

#### The state-trait anxiety inventory (STAI)

The STAI was used to measure both state and trait anxiety. This scale has been widely used, and has demonstrated good reliability and validity in both general and clinical populations (STAI-State alpha = .94, STAI-Trait alpha = .94) [19].

#### The depression anxiety and stress scale short version (DASS-21)

The DASS-21 was used as a secondary measure of anxiety, and to identify those with stress and depression symptoms [20] as it is a widely used clinical screening tool for these constructs and has good reliability (alpha = .89 stress, .90 depression, .79 anxiety) and validity [19].

### Statistical analysis

The data were analysed using SPSS Statistics Version 22. The data were checked for missing values, and items recoded as required. Only one missing value was found for age. Listwise exclusion of the missing value was performed. Statistical assumptions were checked for all relevant analyses, including skewness and kurtosis of variables. Normal distributions were checked using histograms, and boxplots used to check for univariate outliers. All assumptions were found to be within acceptable range.

Descriptive statistics were used to report means, frequencies, and percentages. Chi-square test for goodness of fit was used to determine differences in the frequency of herbal medicine use for anxiety symptoms between those with and without an anxiety disorder diagnosis.

As we found a high amount of non-disclosure of herbal medicine use in our sample, we sought to determine whether there was a relationship between nondisclosure and the following variables: anxiety disorder diagnosis, anxiety symptoms in the previous 12 months, having used herbal medicines for anxiety symptoms, having used pharmaceuticals for anxiety symptoms, and having used a combination of herbal medicines and pharmaceuticals for anxiety symptoms. Chi-square test of contingency was used to test relations between dichotomous variables, and Cramer’s V was used to report effect size [21].

A two-way analysis of variance (ANOVA) was used to examine the effects of anxiety disorder diagnosis and having anxiety symptoms in the previous 12 months on herbal medicine use in the previous 12 months. In addition, simple logistic regression was performed to determine whether anxiety symptoms in the previous week predicted current herbal medicine use for anxiety symptoms (a dichotomous variable).

## Results

### Participant demographics

Of the 400 participants there were 203 males (50.8 %) and 197 females (49.3 %). The mean age was 49.1 (*SD* = 15.53), with ages ranging from 18 to 85. The majority of participants resided in the state of New South Wales (NSW) (34 %), while the least represented state was the Northern Territory (NT) (*n* = 1). The highest level of education for the majority of participants was Bachelor Degree (22.8 %), with the least amount of participants either having a Year 10 or equivalent (12 %) or a Postgraduate qualification (12 %) as their highest level of education. Participants’ demographics are summarised in Table 1.

**Table 1:** Participants’ demographics including: age, gender, state of residence, and education level

Total *N* = 400	
	% (*n*)
Gender	
Female	49.3 (197)
Male	50.8 (203)
Age	
18–30	16 (64)
31–40	14.5 (58)
41–50	19.5 (78)
51–60	22.6 (90)
>60	27.3 (109)
State/Territory	
ACT	2.75 (11)
NSW	34 (133)
NT	0.3 (1)
QLD	14.3 (57)
SA	9.3 (37)
TAS	3.8 (15)
VIC	25.3 (101)
WA	11 (44)
Education level	
Year 10 or equivalent	12 (48)
Year 12 or equivalent	15.8 (63)
Trade certificate	16 (64)
Diploma or Advanced diploma	21.5 (86)
Bachelor degree	22.8 (91)
Postgraduate qualification	12 (48)

*Note*. There was missing data for one participant on the variable age and state/territory. *ACT* Australian Capital Territory, *NSW* New South Wales, *NT* Northern Territory, *QLD* Queensland, *SA* South Australia, *TAS* Tasmania, *VIC* Victoria, *WA* Western Australia

### Scale reliabilities and mental health characteristics of participants

The reliabilities for each of the DASS-21 and the STAI scales in the current study were excellent, and within the range of previous research using similar cohorts. The Cronbach alpha levels are displayed in Table 1 with means and standard deviations, along with reference to previous studies using general population and similar clinical samples. The mean scores for the DASS-21 scales indicate that this cohort has worse mental health compared to general population norms, with stress classified as mild, and depression and anxiety as moderate [22]. The mean scores for the STAI State and Trait scales were between general population and clinical norms, as shown in Table 2.

**Table 2:** Scale reliabilities of the DASS-21 and STAI

	Current study			General population norms^a^			Clinical population		
Scale	M	SD	α	M	SD	α	M	SD	α
DASS-21 Stress	8.07	5.28	.91	3.99	4.24	.89	21.10^b^	11.15^b^	.93^b^
DASS-21 Depression	6.98	5.87	.94	2.57	3.86	.90	10.65^b^	9.30^b^	.96^b^
DASS Anxiety	5.82	5.14	.90	1.74	2.78	.79	10.90^b^	8.12^b^	.89^b^
STAI Trait	46.63	12.39	.94	36.35	11.39	.94	55^c^	10^c^	_^d^
STAI State	43.65	12.87	.94	33.16	11.69	.94	52^c^	13^c^	_ ^d^

*Note*. α Cronbach alpha, *DASS* Depression Anxiety Stress Scale, *STAI* State Trait Anxiety Inventory

^a^ Australian general population norms taken from [19]

^b^ Clinical population norms (anxiety or mood disorder diagnosis) for the DASS-21 taken from [21]

^c^ Clinical population norms (GAD diagnosis) for the STAI State and Trait scales taken from [22]

^d^ Not reported

### Anxiety and medicine use characteristics

The majority of participants (82.3 %) experienced anxiety symptoms in the previous 12 months, with 47 % reporting having previously been diagnosed with an anxiety disorder (type of disorder not specified). In addition, 72.8 % of participants used herbal medicines specifically for anxiety symptoms in their lifetime, while 55.3 % had used prescribed pharmaceuticals, with 27.5 % having used herbal medicines concurrently with prescribed pharmaceuticals. Of those with an anxiety disorder diagnosis (*n* = 188), 83.5 % used herbal medicines for anxiety symptoms, which compared to 63.2 % of those without a disorder diagnosis (*n* = 134). Chi-square test for goodness of fit indicated that those with an anxiety disorder diagnosis reported a significantly greater frequency of herbal medicine use for anxiety symptoms than those without a diagnosis, *χ*2 (1, *N* = 188) = 84.45, *p* = .000. Cohen’s *w* was 0.67, which is a large effect size [21].

### Practitioner use and herbal medicine prescribing

General practitioners were the most frequently consulted health practitioner, with 87 % of people having seen a medical doctor in the last 12 months, with an average of 12 visits over this time. In contrast, Western herbalists (4.8 %) and homeopaths (4.5 %) were the practitioners seen by the least number of participants. However, while it was not common for people to consult Western herbalists, those who did had an average of 7.5 visits in the previous 12 months, which was the second highest number of consultations for a practitioner during that time. The second most consulted practitioners in the previous 12 months were psychologists (19.5 %), who were seen an average of 6.2 times. All health practitioners were reported to have prescribed herbal medicines to varying degrees, with the most frequent being traditional Chinese medicine (TCM) practitioners (83.3 %), Western herbalists (73.7 %), and naturopaths (77 %). This compared to the lowest prescribers of herbal medicines, who were psychiatrists (9.1 %), psychologists (7.7 %), and general practitioners (5.5 %). Health practitioner use and herbal medicine prescription is summarised in Table 3.

**Table 3:** Type of health practitioner seen and prescription of herbal medicines

Practitioner type	Frequency of type of practitioner seen in the previous 12 months^a^	Number of visits in the previous 12 months	Frequency of herbal medicine prescription by practitioner
	% (*n*)	M ^b^	% (*n*)^c^
General practitioner	87 (348)	12.04	5.5 (19)
Psychologist	19.5 (78)	6.20	7.7 (6)
Chiropractor	15 (60)	6.62	15 (9)
Acupuncturist	13 (52)	6.73	42.3 (22)
Naturopath	12 (48)	2.76	77.1 (37)
TCM practitioner	12 (48)	4.83	83.3 (40)
Nutritionist	10.3 (41)	3.07	19.5 (8)
Psychiatrist	8.3 (33)	4.88	9.1 (3)
Western herbalist	4.8 (19)	7.56	73.7 (14)
Homeopath	4.5 (18)	4.00	72.2 (13)
Other	16.8 (67)	8.89	12.1 (8)
Manual therapist	5 (20)		
Medical specialist	4.8 (19)		
CAM practitioner^d^	2.8 (11)		
None	6.8 (27)		

*Note. TCM* Traditional Chinese medicine practitioner. *CAM* Complementary and alternative medicine

^a^A multiple response item; participants were asked to select from a predetermined list of health practitioners or *other* where they could list the other type of practitioner seen

^b^Mean number of visits to practitioner in previous 12 months

^c^Frequency and percentage of number of each practitioner seen in the previous 12 months

^d^Other CAM practitioners included iridologist, reflexologist; and Japanese, Korean, and Indonesian herbalists as described by participants

### Herbal medicine use

The most commonly used herbal medicine to treat anxiety symptoms in the previous 12 months was chamomile (43 %), followed by lavender (32.5 %). For all herbs used self-prescription was common, and ranged from 8.7 % (gotu kola) to 68 % (chamomile) for individual herbs. Ginkgo was reported to be the most frequently prescribed herb (31.3 %) to treat anxiety symptoms by a herbal medicine practitioner, with oats being the least prescribed by these practitioners (5.1 %). The least used herbs for anxiety symptoms in the previous 12 months were zizyphus (1.5 %), bacopa (2 %), and skullcap (2 %). See Table 4 for the frequency of herbal medicines used for anxiety symptoms, and how they were prescribed. Chamomile was most frequently ingested as a tea (89.7 %), with liquorice most frequently used as a powder (59.1 %), valerian most frequently used as a tablet (70.5 %), and lavender most frequently used as a liquid extract (34.3 %). All herbal medicines were used in a range of different preparations (see Table 5).

**Table 4:** Frequency of herbal medicines used to treat anxiety symptoms and who prescribed each medicine

Herbal medicine	Use in previous 12 months (*N* = 400)	Current use^a^	Who prescribed % (*n*)						
	% (*n*)	% (*n*)	Self-prescribed	Herbal medicine practitioner	Medical doctor/psychiatrist	Shop assistant	Pharmacist	Other CAM	Other
Bacopa	2 (8)	62.5 (5)	35.7 (5)	7.1 (1)	7.1 (1)	0 (0)	0 (0)	0 (0)	50 (7)
Chamomile	43 (172)	65.1 (112)	68 (119)	15.4 (27)	1.7 (3)	4 (7)	2.9 (5)	3.4 (6)	4.6 (8)
Ginkgo	13.8 (55)	47.3 (26)	42.2 (27)	31.3 (20)	3.1 (2)	3.1 (2)	0 (0)	3.1 (2)	17.2 (11)
Ginseng^b^	19.5 (78)	50 (39)	37.1 (33)	28.1 (25)	3.4 (3)	5.6 (5)	3.4 (3)	3.4 (3)	19.1 (17)
Gotu kola	1.5 (6)	83.3 (5)	8.7 (2)	17.4 (4)	0 (0)	0 (0)	0 (0)	0 (0)	73.9 (17)
Kava	3.5 (14)	42.9 (6)	16.7 (5)	20 (6)	6.7 (2)	0 (0)	0 (0)	0 (0)	56.7 (17)
Lavender	32.5 (130)	60.8 (79)	58.6 (82)	12.9 (18)	5 (7)	4.3 (6)	1.4 (2)	2.1 (3)	15.7 (22)
Lemon balm	8.3 (33)	57.6 (19)	32.7 (16)	22.4 (11)	2 (1)	0 (0)	0 (0)	4.1 (2)	38.8 (19)
Liquorice	11 (44)	43.2 (19)	50.8 (30)	6.8 (4)	3.4 (2)	5.1 (3)	5.1 (3)	1.7 (1)	27.1 (16)
Oats	15.5 (62)	59.7 (37)	55.1 (43)	5.1 (4)	5.1 (4)	7.7 (6)	0 (0)	3.8 (3)	23.1 (18)
Passionflower	7 (28)	60.7 (17)	29.4 (15)	15.7 (8)	7.8 (4)	2 (1)	0 (0)	0 (0)	45.1 (23)
Rhodiola	3 (12)	58.3 (7)	10.8 (4)	16.2 (6)	2.7 (1)	0 (0)	0 (0)	2.7 (1)	67.6 (25)
Skullcap	2 (8)	37.5 (3)	9.4 (3)	6.3 (2)	6.3 (2)	0 (0)	0 (0)	3.1 (1)	72 (24)
St John’s wort	19.3 (77)	39 (30)	36 (36)	23 (23)	4 (4)	6 (6)	6 (6)	2 (2)	23 (23)
Valerian	20.8 (83)	45.8 (38)	45.7 (48)	13.3 (14)	7.6 (8)	5.7 (6)	1.9 (2)	1.9 (2)	23.8 (25)
Withania	2.5 (10)	60 (6)	11.4 (4)	11.4 (4)	0 (0)	2.9 (1)	0 (0)	2.9 (1)	71.4 (25)
Zizyphus	1.5 (6)	83.3 (5)	9.7 (3)	9.7 (3)	0 (0)	0 (0)	0 (0)	0 (0)	80.6 (25)
Other	19.8 (79)	40 (32)	26.9 (28)	8.7 (9)	9.6 (10)	3.8 (4)	0 (0)	3.8 (4)	47.1 (49)

^a^Reported as percentage of users of each herb in the previous 12 months

^b^ Either Koren or Siberian ginseng

^c^ Herbal medicine practitioner included naturopaths, Western herbalists, traditional Chinese medicine practitioners, and acupuncturists

^d^Other CAM = includes chiropractors, nutritionists, and homeopaths

^e^Other general = any other health practitioner not listed

**Table 5:** Percentage of users for each herbal medicine preparation

Herbal medicine	Herbal medicine preparation type % (*n*)^a^					
	Tea	Powder	Tablet	Liquid extract	In a formula	Other
Bacopa	21.4 (3)	0 (0)	14.3 (2)	14.3 (2)	50 (4)	50 (7)
Chamomile	89.7 (157)	1.7 (3)	3.4 (6)	3.4 (6)	25.6 (44)	1.7 (3)
Ginkgo	32.8 (21)	7.8 (5)	37.5 (24)	7.8 (5)	56.4 (31)	14.1 (9)
Ginseng (Korean or Siberian)	30.3 (27)	7.9 (7)	33.7 (30)	13.5 (12)	43.6 (34)	14.6 (13)
Gotu kola	8.7 (2)	4.3 (1)	4.3 (1)	4.3 (1)	66.7 (4)	78.3 (18)
Kava	16.7 (5)	6.7 (2)	16.7 (5)	3.3 (1)	35.7 (5)	56.7 (17)
Lavender	20 (28)	7.9 (11)	3.6 (5)	34.3 (48)	23.8 (31)	34.3 (48)
Lemon balm	44.9 (22)	6.1 (3)	4.1 (2)	12.2 (6)	42.4 (14)	32.7 (16)
Liquorice	40.9 (18)	59.1 (26)	0 (0)	0 (0)	36.4 (16)	0 (0)
Oats	10.3 (8)	28.2 (22)	7.7 (6)	10.3 (8)	32.3 (20)	43.6 (34)
Passionflower	23.5 (12)	3.9 (2)	19.6 (10)	7.8 (4)	71.4 (20)	45.1 (23)
Rhodiola	8.1 (3)	5.4 (2)	16.2 (6)	2.7 (1)	75 (9)	67.6 (25)
Skullcap	9.4 (3)	3.1 (1)	3.1 (1)	6.3 (2)	62.5 (5)	78.1 (25)
St John’s wort	6 (6)	1 (1)	61 (61)	9 (9)	33.8 (26)	23 (23)
Valerian	4.8 (5)	1 (1)	70.5 (74)	2.9 (3)	33.7 (28)	21 (22)
Withania	14.3 (5)	8.6 (3)	5.7 (2)	0 (0)	60 (6)	71.4 (25)
Zizyphus	3.2 (1)	0 (0)	9.7(3)	3.2 (1)	66.7 (4)	83.9 (26)
Other	14.4 (15)	0 (0)	19.2 (20)	6.7 (7)	17.7 (14)	59.6 (62)

^a^Reported as percentage of users of each herb in previous 12 months

### Information sources

The Internet (53 %) was the most frequently used source of information for herbal medicines, with media advertisements being used the least (5.5 %). Use of information sources is displayed in Table 6.

**Table 6:** Frequency of use of herbal medicine information sources

Information sources	Frequency of use
	% (*n*)^a^
The Internet	53 (212)
Friend or family	43.8 (175)
Health food shop assistant	24.8 (99)
Herbal medicine practitioner	21.8 (87)
General practitioner	21.5 (86)
Magazines/newspapers	17.5 (70)
Pharmacist	17.3 (69)
Pharmacy assistant	13.8 (55)
Other health care provider	9.5 (38)
Other	9.5 (38)
Media advertising	5.5 (22)

^a^Multiple response item reported as percent of cases

### Disclosure of herbal medicine use

Forty eight percent of participants did not disclose their herbal medicine use to their doctor, with 55.3 % not disclosing to other health care providers.

Disclosure of herbal medicine use to both medical doctors and other health practitioners had small significant relations with the following variables: having had an anxiety disorder diagnosis, having used herbal medicines for anxiety symptoms, having used pharmaceutical medicines for anxiety symptoms, and having used a combination of pharmaceutical and herbal medicines for anxiety symptoms. The effect size of all these relations was small according to Cramer’s V [21]. These results mean that people who had an anxiety disorder diagnosis, or used either herbal medicines or pharmaceuticals for anxiety symptoms, were significantly more likely to disclose their herbal medicine use to their doctor and other health practitioners. However, those who used a combination of herbal and pharmaceutical medicines for anxiety symptoms were significantly less likely to disclose their use. There was no relation found between disclosure of herbal medicine use and having anxiety symptoms in the previous 12 months. Statistics and results for the chi-square tests of contingency are presented in Table 7.

**Table 7:** Disclosure of herbal medicine use to doctor and other health practitioners

	Disclosure of herbal medicine use to doctor (*N* = 400, *df* = 1)						Disclosure of herbal medicine use to other health practitioners (*N* = 400, *df* = 1)					
	No % (*n*)	Yes % (*n*)	Total % (*n*)	Chi square	*p*	Cramer’s V	No % (*n*)	Yes % (*n*)	Total % (*n*)	Chi square	*p*	Cramer’s V
Anxiety disorder diagnosis												
Yes	*43.8 (91)*	*56.3 (117)*	47 (188)				46.3 (87)	53.7 (101)	47 (188)			
No	63 (121)	37 (71)	53 (212)				63.2 (134)	36.8 (78)	53 (212)			
Total	48 (192)	52 (208)	100 (400)	14.88**	.000	.19	55.3 (221)	44.8 (179)	100 (400)	11.55**	.001	.17
Anxiety symptoms in previous 12 months												
Yes	15.9 (33)	84.1 (175)	82.3 (329)				54.1 (178)	45.9 (151)	82.3 (329)			
No	19.8 (38)	80.2 (154)	17.8 (71)				60.6 (43)	39.4 (28)	17.8 (71)			
Total	48 (192)	52 (208)	100 (400)	1.05	.305		55.3 (221)	44.8 (179)	100 (400)	.99	.321	
Used herbal medicines for anxiety symptoms												
Yes	20.7 (43)	79.3 (165)	72.8 (291)				48.5 (141)	51.5 (150)	72.8 (291)			
No	34.4 (66)	65.6 (126)	27.3 (109)				73.4 (80)	26.6 (29)	27.3 (109)			
Total	48 (192)	52 (208)	100 (400)	9.46**	.002	.15	55.3 (221)	44.8 (179)	100 (400)	19.96**	.000	.22
Used pharmaceuticals for anxiety symptoms												
Yes	35.1 (73)	64.9 (135)	55.3 (221)				48.4 (107)	51.6 (114)	55.3 (221)			
No	52.5 (106)	44.8 (86)	44.8 (179)				63.7 (114)	36.3 (65)	44.8 (179)			
Total	48 (192)	52 (208)	100 (400)	16.33**	.000	.20	55.3 (221)	44.8 (179)	100 (400)	9.39**	.002	.15
Combined pharmaceuticals with herbal medicines for anxiety symptoms												
Yes	66.8 (139)	33.2 (69)	27.5 (110)				40.9 (45)	59.1 (65)	27.5 (110)			
No	78.6 (151)	21.5 (41)	72.5 (290)				60.7 (176)	39.3 (114)	72.5 (290)			
Total	48 (192)	52 (208)	100 (400)	6.70**	.008	.13	55.3 (221)	44.8 (179)	100 (400)	12.62**	.000	.18

** < *p* = 0.01

*Note*. Chi-square test of contingency tested relations between dichotomous variables

### Anxiety severity and herbal medicine use for anxiety symptoms

We further investigated the characteristics of those who used herbal medicines for anxiety symptoms. First, we were interested in whether having anxiety symptoms in the previous 12 months or an anxiety disorder diagnosis, influenced the probability of herbal medicine use in the previous 12 months. Second, we wanted to know if anxiety symptom severity in the previous week predicted current herbal medicine use for anxiety symptoms.

A two-way analysis of variance (ANOVA) was used to examine the effects of anxiety disorder diagnosis and having anxiety symptoms in the previous 12 months on herbal medicine use in the previous 12 months. There was homogeneity of variances as assessed by Levene’s test, *p* = .45. People with a previous anxiety disorder diagnosis (*M* = 4.90, *SE* = 0.25, 95 % CI [4.40, 5.40]) were more likely to use herbal medicines in the previous 12 months than those without an anxiety disorder diagnosis (*M* = 4.04, *SE* = 0.18, 95 % CI [3.70, 4.39]). Anxiety disorder diagnosis accounted for 2 % of the variability of herbal medicine use in the previous 12 months, which is a small effect size (partial η^2^ = .02). Anxiety symptoms in the previous 12 months accounted for 2 % of the variability in herbal medicine use for anxiety symptoms in the previous 12 months, partial η^2^ = .02 (see Table 8).

**Table 8:** Anxiety disorder diagnosis and anxiety symptoms in previous 12 months predicting herbal medicine use

Variables	*df*	*F*	*SS*	*p*
Anxiety disorder diagnosis	1, 396	7.77	36.78	.006**
Anxiety symptoms in previous 12 months	1, 396	9.66	45.75	.002**
AD x AS	1, 396	2.54	12.05	.112

** < *p* = 0.01

*Note. AD* anxiety disorder diagnosis, *AS* anxiety symptoms. A two-way analysis of variance (ANOVA) examined the effects of anxiety disorder diagnosis and having anxiety symptoms in previous 12 months on herbal medicine use in the previous 12 months

People with anxiety symptoms in the previous 12 months (*M* = 4.95, *SE* = 0.12, 95 % CI [4.71, 5.19]) were significantly more likely to use herbal medicines for anxiety symptoms in the previous 12 months than those without anxiety symptoms (*M* = 3.99, *SE* = 0.28, 95 % CI [3.44, 4.55]). Anxiety symptoms in the previous 12 months accounted for 2 % of the variability of herbal medicine use in the previous 12 months, which is a small effect size, partial η^2^ .02. See Table 8 for results of the two-way ANOVA. There was no interaction between anxiety disorder diagnosis and anxiety symptoms in the previous 12 months.

Simple logistic regression showed that severity of anxiety symptoms in the previous week statistically significantly predicted 6 % (Nagelkerke *R*^2^) of the variance in current herbal medicine use for anxiety symptoms, *χ*^2^ = 18.03, and correctly classified 63.5 % of cases. The results and odds ratio are presented in Table 9, which suggest that the odds of currently using herbal medicines for anxiety symptoms are significantly greater for people with more severe anxiety symptoms.

**Table 9:** Logistic regression predicting likelihood of current herbal medicine use for anxiety symptoms

Variable	*B*	*SE*	Wald	*df*	*p*	Odds Ratio	95 % CI for Odds Ratio
Anxiety symptoms	.09	.02	16.20	1	.00	1.10	[1.05, 1.15]

## Discussion

This is the first known study to provide a comprehensive description of how Australian adults who experience anxiety symptoms are using herbal medicines. Almost half the participants had previously been diagnosed with an anxiety disorder, with the remaining having experienced anxiety symptoms. The majority (73 %) used herbal medicines specifically for their anxiety symptoms, with half the cohort having experienced moderate to severe anxiety symptoms in the previous week. These results support previous research finding that Australian adults are using herbal medicines for anxiety symptoms [23].

Participants had consultations with a range of health professionals. Medical doctors were the most frequently consulted health practitioners in the previous 12 months, but they did not prescribe herbal medicines often. This contrasted with Western herbalists who were one of the least consulted practitioners, who prescribed herbal medicines most frequently. The low frequency of consultations with Western herbalists is consistent with previous research including Australian woman [24]. Western herbalists are unregulated in Australia and not considered part of the conventional health system [13], which may partly explain why so few people reported consulting these practitioners. However, in the current study consultations to naturopaths (who are also unregulated) were reported to be as frequent as TCM practitioners who are regulated, suggesting other factors are involved. Some practitioners specialise in Western herbal medicine, but advertise themselves as other types of practitioners; for example, many naturopaths specialise in Western herbal medicine. This may cause confusion for health care consumers in terms of being able to accurately identify what type of practitioner they are consulting, which may have affected the accuracy of the reporting of these consults. Despite the low number of participants consulting Western herbalists they were reported to have the second highest number visits in the previous 12 months for a practitioner. It is unclear why this is the case.

Practitioners with specialised training in the clinical prescription of herbal medicines (i.e. Western herbalists, TCM practitioners, and naturopaths) were reported to prescribe herbal medicines more frequently than other practitioners (e.g. medical doctors and psychologists). This is unsurprising given that other health practitioners do not receive undergraduate training in herbal medicine prescribing; therefore, may either lack confidence or ability to prescribe these medicines, be unaware of the efficacy and safety of herbal medicines, or choose other treatments that they consider more appropriate, such as pharmaceuticals and psychological interventions.

A range of herbal medicines were being used to treat anxiety symptoms, with variation in the preparation types used for each herb. This is likely to relate to factors such as taste, availability of the type of preparation, who is prescribing or selling the herbal medicine, and cultural use. For example, chamomile was the most frequently used herbal medicine for anxiety symptoms, the most frequently self-prescribed, and most frequently consumed as a tea. The high rates of self-prescription of chamomile is likely to reflect this herb being widely available, and commonly being consumed as a beverage for enjoyment. In contrast, valerian and St John’s wort were also frequently self-prescribed, however they are more commonly available as a tablet, which was the preparation most frequently used for these herbs.

High amounts of self-prescribing of herbal medicines was found in this cohort (up to 68 %), which is consistent with self-prescribing previously reported in the Australian general population (51.8 %) [23], and more recently in women during pregnancy (77.9 %) [15]. In contrast, there was less practitioner prescribing reported, with only up to 31.3 % of individual herbs being prescribed by herbal medicine practitioners, which is slightly higher than previously reported in the general Australian population (27.4 %) [23]. People with anxiety disorders have been found to have barriers to accessing health care, such as lack of time, treatment cost, embarrassment, and stigma [25], which may partly explain the high rates of self-prescribing of herbal medicines in our study. In addition, people with mental health problems have reported preference to self-manage their symptoms [26]. We also found that 48 % of participants did not disclose their herbal medicine use to their treating medical doctor. This is consistent with research in psychiatric outpatients finding that between 37 [16] and 49 % [27] of patients did not disclose their CAM use to their healthcare providers. In addition, our study found that those using herbal medicines concurrently with pharmaceuticals were less likely to disclose their use. Over 27 % of participants had used herbal medicines concurrently with pharmaceuticals for their anxiety symptoms, which is consistent with CAM users in an Australian population sample (26 %) [23]. However, in our study the people who had an anxiety disorder diagnosis, or used pharmaceutical medicines for anxiety symptoms were more likely to disclose their herbal medicine use to their medical doctor. It is difficult to identify why these people are more likely to disclose herbal medicine use as it contradicts findings from research mentioned previously. Perhaps they are more comfortable with discussing their anxiety and other issues with their health practitioners as they have already been prescribed a medication for their anxiety symptoms and may not be concerned about the stigma, or they have an established rapport with their health care providers and feel supported to disclose their use.

Self-prescription and non-disclosure of herbal medicine use is problematic as it does not allow for health practitioners to provide their patients with optimal care, and there is the potential for herb-drugs interactions. For example, kava (*Piper methysticum*)*,* gingko (*Gingko biloba*), St John’s wort (*Hypericum perforatum*) and ginseng (*Panax ginseng)*—all used by participants in our study—have been found to interact with various classes of commonly prescribed pharmaceuticals [12]. The reasons for these behaviours are unclear, however it may be that those who are combining herbal medicines with pharmaceuticals are concerned about disclosing this to their health care providers for fear of discrimination. Discrimination in health care was found to be related to self-prescribed herbal medicine use in a representative sample of US adults [28]. In addition, being dissatisfied with the medical encounter has been found to predict CAM use in both cancer [29, 30] and general population samples [31, 32]. More research is needed in this area to determine the reasons for discrimination by health practitioners, especially considering that people with mental health problems report stigma as a barrier to health care [5].

Herbs with evidence of efficacy in treating anxiety (i.e. kava and passionflower) [33] were not frequently used in this study, suggesting that people may not receive information about the efficacy of these herbal medicines. This is consistent with a US study finding that people were not using herbal medicines according to their evidence-based indications [14]. This may be related to our finding that non-professional information sources, such as the Internet and family and friends, were the most frequently used when making decisions about herbal medicine use. High use of non-professional information sources has previously been found in psychiatric patients [16] and other at risk cohorts, such as pregnant women [15]. It is not clear why people use non-professional information sources. However, qualitative interviews found that people with anxiety value their own experience and that of important others as a form of evidence [34]. This is an important behaviour to understand, and needs more research.

Almost half the participants were currently taking herbal medicines for anxiety symptoms while not experiencing problematic anxiety symptoms in the previous week. It is unclear why they were taking these medicines. However, herbal medicines could be effective in providing some relief of anxiety symptoms, or are being taken as a preventative treatment. Maintaining health and preventing illness [16] have previously been identified as reasons for taking herbal medicines. Zhang and colleagues [23] reported that over 50 % of Australian adults who took commonly used herbs did so to enhance their health. The use of herbal anxiolytics when experiencing less severe anxiety may potentially prevent subthreshold anxiety from developing into a more serious disorder. This is an area for future research.

We found that people with an anxiety disorder diagnosis were more likely to use herbal medicines than those without. This is consistent with previous research reporting higher amounts of herbal medicine use in people with an anxiety disorder diagnosis [8]. We also found that people with more severe anxiety symptoms in the previous week were more likely to use herbal medicines for anxiety symptoms compared to those without, which suggests that anxiety severity increases the use of herbal medicines for anxiety symptoms. As over 30 % of people with anxiety disorders do not respond to evidence-based psychological and pharmaceutical treatments [4], they may seek additional relief for anxiety symptoms, which could be one reason why people are taking herbal medicines concurrently with pharmaceuticals. Some people may also be using psychological treatments at the same time as taking herbal medicines. Participants were not asked about their use of psychological therapies in this study. Future research could seek to determine the full range of treatments people are using for their anxiety symptoms and reasons for choosing these therapies.

In respect to study limitations, this study relied on self-report of previous herbal medicine use and anxiety disorder diagnosis, which may be affected by recall bias. As a convenience sample was used the generalisability of the results is limited due to potential response bias. In addition, the prevalence of herbal medicine use for anxiety symptoms in this cohort cannot be generalised to the broader population. However, the sample demographics suggest that the sample was reasonably representative of the Australian population with regards to gender [35], and geographic location [36], but slightly older than the median population age [35]. This study does provide a comprehensive description of herbal medicine use behaviour in a sample of adults who experience anxiety. More research on specific population samples is needed to determine prevalence rates of herbal medicine use for anxiety.

Future research should seek to understand decision making involved in using herbal medicines for anxiety symptoms. In certain instances self-prescription can be done safely; however, it can be difficult for the public to assess the information presented to them [37]. In addition, it is difficult to determine if people are receiving the most suitable treatment for their needs, as they are self-prescribing, consulting with a range of health practitioners, and not disclosing their herbal medicine use. Therefore, it needs to be determined how accurate reliable information can be made more accessible to both the public and health practitioners, so they can be better educated about the efficacy, effectiveness, and safety of herbal anxiolytics to ensure the most suitable treatments are used. As medical doctors were the most frequently seen health care provider in our study it is critical that they continue to educate themselves and their patients about herbal medicine.

## Conclusions

This study is the first to measure how a cohort of Australian adults with an experience of anxiety are using herbal medicines. Herbal medicines are being used by adults with anxiety and are commonly self-prescribed for anxiety symptoms. Health practitioners who are experts in herbal medicine prescribing are consulted infrequently. In addition, herbal medicine use is often not disclosed to health practitioners. These behaviours are concerning as people may not be receiving the most suitable treatments for their needs, and may even be dangerous. These findings can inform future research to assist in determining the reasons for non-disclosure, and high rates of self-prescribing. Given that herbal medicine use and self-prescription in the general population is increasing, it is critical we develop a better understanding of why people are using these medicines, and how we can develop improved health literacy to help with treatment decision making to ensure they receive optimal care.

## Abbreviations

CAM: Complementary and alternative medicine
DASS: Depression, Anxiety and Stress Scale
STAI: State Trait Anxiety Inventory
TCM: Traditional Chinese medicine

## References

[1] Slade T, Johnston A, Oakley Browne MA, Andrews G, Whiteford H (2007). National Survey of Mental Health and Wellbeing: methods and key findings. Aust N Z J Psychiatry.

[2] Kessler RC, Wittchen H-U (2002). Patterns and correlates of generalized anxiety disorder in community samples. J Clin Psychiatry.

[3] Grenier S, Préville M, Boyer R, O’Connor K, Béland S-G, Potvin O (2011). The impact of DSM-IV symptom and clinical significance criteria on the prevalence estimates of subthreshold and threshold anxiety in the older adult population. Am J Geriatr Psychiatry.

[4] Taylor S, Abramowitz JS, McKay D (2012). Non-adherence and non-response in the treatment of anxiety disorders. J Anxiety Disord.

[5] Clement S, Schauman O, Graham T, Maggioni F, Evans-Lacko S, Bezborodovs N (2014). What is the impact of mental health-related stigma on help-seeking? A systematic review of quantitative and qualitative studies. Psychol Med.

[6] Thompson P, Jones J, Evans J, Leslie SJ (2012). Factors influencing the use of complementary and alternative medicine and whether patients inform their primary care physician. Complement Ther Med.

[7] McIntyre E, Saliba AJ, Wiener K, Sarris J (2015). Prevalence and predictors of herbal medicine use in adults experiencing anxiety: a critical review of the literature. Advances in Integrative Medicine.

[8] Ravven SE, Zimmerman MB, Schultz SK, Wallace RB (2011). 12-month herbal medicine use for mental health from the National Comorbidity Survey Replication (NCS-R). Ann Clin Psychiatry.

[9] Bystritsky A, Hovav S, Sherbourne C, Stein MB, Rose RD, Campbell-Sills L (2012). Use of complementary and alternative medicine in a large sample of anxiety patients. Psychosomatics.

[10] Bahceci B, Bagcioglu E, ÖZTÜRK A, Bulbul F, Sahiner IV, Tuncer BE (2013). Complementary and alternative medicine use in patients with mental disorders in Turkey. Complement Ther Clin Pract.

[11] Wahlström M, Sihvo S, Haukkala A (2008). Use of mental health services and complementary and alternative medicine in persons with common mental disorders. Acta Psychiatr Scand.

[12] Posadzki P, Watson L, Ernst E (2013). Herb-drug interactions: an overview of systematic reviews. Br J Clin Pharmacol.

[13] Wardle JJL, Adams J (2014). Indirect and non-health risks associated with complementary and alternative medicine use: an integrative review. Eur J Integr Med.

[14] Bardia A, Nisly NL, Zimmerman MB, Gryzlak BM, Wallace RB (2007). Use of herbs among adults based on evidence-based indications: findings from the National Health Interview Survey. Mayo Clin Proc.

[15] Frawley J, Adams J, Steel A, Broom A, Gallois C, Sibbritt D (2015). Women’s use and self-prescription of herbal medicine during pregnancy: an examination of 1,835 pregnant women. Womens Health Issues.

[16] Alderman CP, Kiepfer B (2003). Complementary medicine use by psychiatry patients of an Australian hospital. Ann Pharmacother.

[17] Robinson A, McGrail MR (2004). Disclosure of CAM use to medical practitioners: a review of qualitative and quantitative studies. Complement Ther Med.

[18] Quandt SA, Verhoef MJ, Arcury TA, Lewith GT, Steinsbekk A, Kristoffersen AE (2009). Development of an international questionnaire to measure use of complementary and alternative medicine (I-CAM-Q). J Altern Complement Med.

[19] Crawford J, Cayley C, Lovibond PF, Wilson PH, Hartley C (2011). Percentile norms and accompanying interval estimates from an Australian general adult population sample for self-report mood scales (BAI, BDI, CRSD, CES-D, DASS, DASS-21, STAI-X, STAI-Y, SRDS, and SRAS). Aust Psychol.

[20] Lovibond PF, Lovibond SH (1995). The structure of negative emotional states: comparison of the Depression Anxiety Stress Scales (DASS) with the Beck Depression and Anxiety Inventories. Behav Res Ther.

[21] Cohen J (1992). A power primer. Psychol Bull.

[22] Lovibond SH, Lovibond PF (1996). Manual for the Depression Anxiety Stress Scales.

[23] Zhang AL, Story DF, Lin V, Vitetta L, Xue CC (2008). A population survey on the use of 24 common medicinal herbs in Australia. Pharmacoepidemiol Drug Saf.

[24] Adams J, Sibbritt D, Young AF (2007). Consultations with a naturopath or herbalist: the prevalence of use and profile of users amongst mid-aged women in Australia. Public Health.

[25] Prins MA, Verhaak PFM, Bensing JM, van der Meer K (2008). Health beliefs and perceived need for mental health care of anxiety and depression—The patients’ perspective explored. Clin Psychol Rev.

[26] Olesen SC, Butterworth P, Leach L (2010). Prevalence of self-management versus formal service use for common mental disorders in Australia: findings from the 2007 National Survey of Mental Health and Wellbeing. Aust N Z J Psychiatry.

[27] Knaudt PR, Connor KM, Weisler RH, Churchill LE, Davidson JR (1999). Alternative therapy use by psychiatric outpatients. J Nerv Ment Dis.

[28] Thorburn S, Faith J, Keon KL, Tippens KM (2013). Discrimination in health care and CAM use in a representative sample of U.S. adults. J Altern Complement Med.

[29] Paltiel O, Avitzour M, Peretz T, Cherny N, Kaduri L, Pfeffer RM (2001). Determinants of the use of complementary therapies by patients with cancer. J Clin Oncol.

[30] Shumay DM, Maskarinec G, Gotay CC, Heiby EM, Kakai H (2002). Determinants of the degree of complementary and alternative medicine use among patients with cancer. J Altern Complement Med.

[31] Sirois FM, Gick ML (2002). An investigation of the health beliefs and motivations of complementary medicine clients. Soc Sci Med.

[32] Siahpush M (1998). Postmodern values, dissatisfaction with conventional medicine and popularity of alternative therapies. J Sociol.

[33] Sarris J, McIntyre E, Camfield DA (2013). Plant-based medicines for anxiety disorders, Part 2: a review of clinical studies with supporting preclinical evidence. CNS Drugs.

[34] McIntyre E, Saliba AJ, Moran CC. Herbal medicine use in adults experiencing anxiety: a qualitative exploration. Int J Qual Stud Health Well-being. 2015;10:29275. 10.3402/qhw.v10.29275 PMC4683991 26680418

[35] Australian Bureau of Statistics (2015). 3235.0 - Population by Age and Sex, Regions of Australia, 2014.

[36] Australian Bureau of Statistics (2015). 3101.0 - Australian Demographic Statistics, Mar 2015.

[37] Shreffler-Grant J, Weinert C, Nichols E (2014). Instrument to measure health literacy about complementary and alternative medicine. J Nurs Measure.

